# Advancements in cobalt‐based oxide catalysts for soot oxidation: Enhancing catalytic performance through modification and morphology control

**DOI:** 10.1002/smo.20240024

**Published:** 2024-10-29

**Authors:** Tingyi Zhao, Zuguo Song, Chengchun Wu, Yuanjun Li, Haoze Li, Yuechang Wei, Siyu Yao, Menglan Xiao, Mingqin Zhao, Bing Cui

**Affiliations:** ^1^ Flavors and Fragrance Engineering & Technology Research Center of Henan Province College of Tobacco Science Henan Agricultural University Zhengzhou China; ^2^ China Tobacco Shaanxi Industrial Co., Ltd. Xian China; ^3^ State Key Laboratory of Heavy Oil Processing Key Laboratory of Optical Detection Technology for Oil and Gas China University of Petroleum Beijing China

**Keywords:** cobalt‐based oxide catalysts, intrinsic activity, morphology, soot oxidation

## Abstract

The widespread use of diesel engines results in significant environmental contamination due to emitted pollutants, particularly soot particles. These pollutants are detrimental to public health. At present, one of the most effective ways to remove soot particles is the catalytic diesel particulate filter after‐treatment technology, which requires the catalyst to have superior low temperature activity. Compared with cerium oxide which is widely used, cobalt oxide in transition metal oxides has been widely studied in recent years because of its high redox ability and easy to control morphology. This paper elaborates on the influence of modification techniques such as doping, loading, and solid solution on the catalytic performance of cobalt‐based catalysts in soot oxidation. Along the same lines, it further reviews the research progress on cobalt‐based oxide catalysts with specific dimensional structures and morphologies in soot oxidation. Finally, it provides an outlook on the challenges faced by the theoretical basis and applied research of cobalt‐based catalysts in soot oxidation.

## Graphical abstract

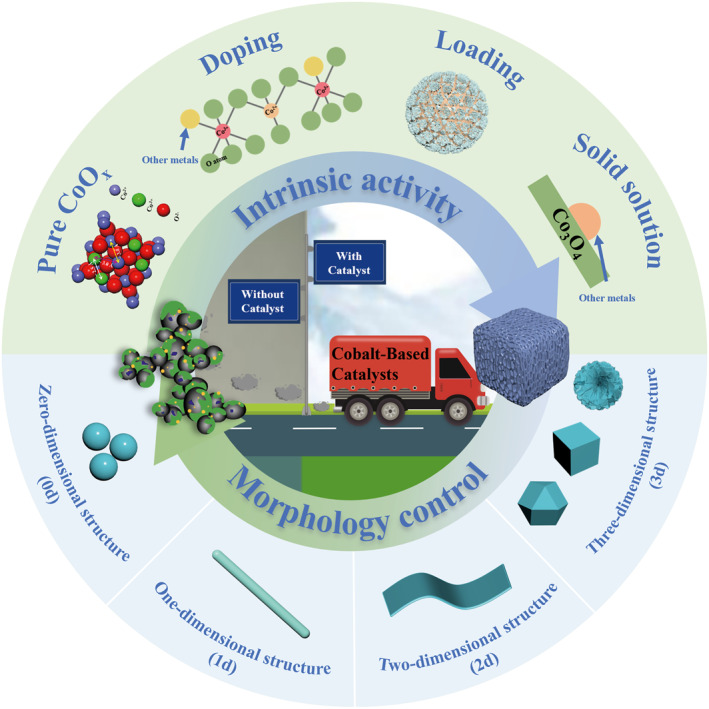

## 1 INTRODUCTION

**FIGURE 1:**
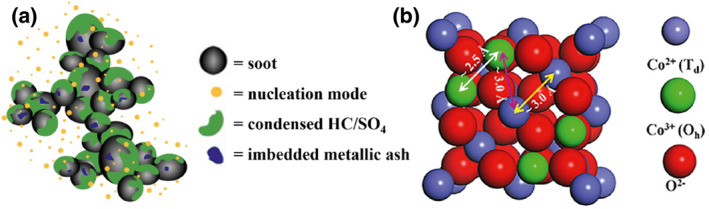
(a) Schematic of diesel particulate matter (PM) composition.^[3]^ Reproduced with permission.^[3]^ Copyright 2007, Elsevier. (b) Relation of interatomic distance between O_h_ and T_d_ atoms in the Co‐based spinel structure.^[4]^ Reproduced with permission.^[4]^ Copyright 2017, ACS Publications.

Due to its high durability, reliability, and fuel efficiency, diesel engines are extensively used in various mobile applications. However, this widespread use also brings about significant environmental pollution issues, primarily through its exhaust emissions.[^1^, ^2^] Extensive studies have been conducted to investigate the composition of particulate matter (PM) emitted from diesel engines. Figure 1a illustrates that PM from diesel exhaust is a complex mixture consisting of soot particles, soluble organic matter (SOF), sulfate, ash, and various other components. Notably, soot particles are highlighted as especially harmful to public health and ecological equilibrium.[^5^, ^6^, ^7^, ^8^] Originating from incomplete combustion of hydrocarbon fuels, these fine and ultrafine particles can easily infiltrate the respiratory system, accumulating within the human body and causing damage to the respiratory and pulmonary systems, leading to respiratory and pulmonary diseases, and even exacerbating cardiovascular diseases and lung cancer.[^9^, ^10^, ^11^] Moreover, soot particles have a prolonged residence time in the atmosphere, directly impacting air quality and potentially exacerbating global climate change to some extent, affecting plant growth, polluting water sources, degrading soil quality, and even jeopardizing biodiversity.^[12]^ To safeguard the environment and human health, effective measures must be taken to mitigate soot emissions.

Catalytic diesel particulate filter (CDPF) is considered an effective after‐treatment technology for controlling soot pollution.^[13]^ The core of this technology is the preparation of catalysts with low temperature activity.^[14]^ In recent years, researchers have reported numerous highly efficient catalysts, which encompass a range of materials, including precious metals,^[15]^ transition metal oxides of group,[^16^, ^17^, ^18^] alkaline metal oxides of group,[^19^, ^20^] perovskite type oxides,[^21^, ^22^] and rare earth oxides.^[23]^ Therein, platinum group metals such as platinum, palladium, rhodium, ruthenium, and iridium have demonstrated excellent performance in catalytic reactions, thus making them extensively utilized in commercial applications.[^24^, ^25^] However, the high cost and relatively low abundance of these elements limit their widespread application. To address this issue, researchers have turned to transition metal catalysts as a potential catalyst main phase material. As a kind of transition metal oxide catalysts, cobalt‐based oxide catalysts (CoO_*x*_) can replace or reduce the use of precious metals to a certain extent, and have found wide applications in catalytic oxidation. The unit cell of Co_3_O_4_ is illustrated in Figure 1B, Co^2+^ ions occupy 1/8 of the tetrahedral voids, each coordinated with four lattice oxygen atoms, while Co^3+^ ions occupy 1/2 of the octahedral voids, each coordinated with six lattice oxygen atoms.^[4]^ Due to the multivalence of cobalt and the synergistic effect of Co^3+^ and Co^2+^, cobalt‐based oxide catalysts exhibit high catalytic performance. By developing and optimizing cobalt‐based oxide catalysts, the cost of catalytic reactions can be reduced, facilitating their industrial application and providing more economical and environmentally friendly solutions for sustainable development.

**FIGURE 2:**
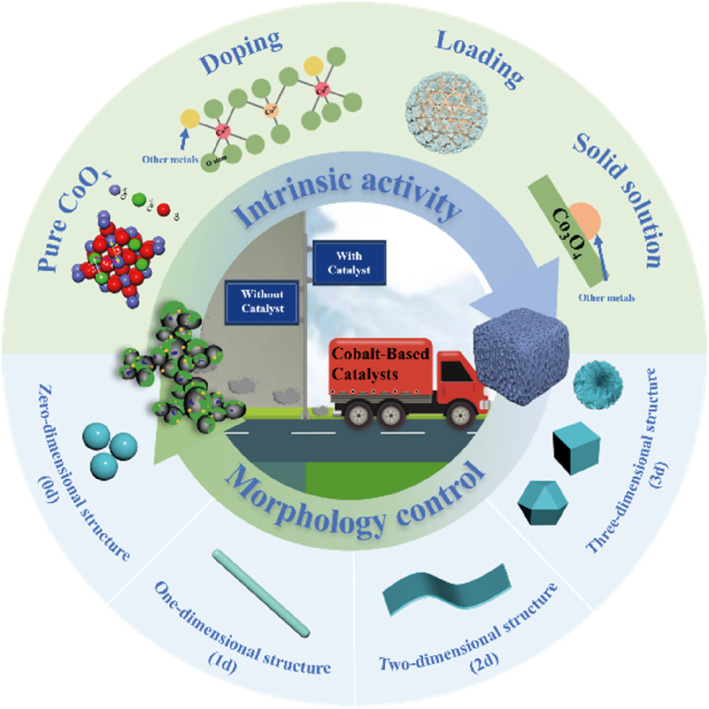
Schematic diagram of cobalt‐based catalysts in the soot catalytic oxidation.

The catalytic oxidation of soot involves a reaction between gas (O_2_), solid (soot), and solid (catalyst). This reaction occurs at a three‐phase contact interface involving the soot and the catalyst, as well as the gaseous reactant and the catalyst.[^26^, ^27^] Previous studies have indicated that the improvement in catalytic performance is related to both intrinsic activity (the inherent redox properties) and solid‐solid contact (the contact between the catalyst and soot particles).^[28]^ Firstly, factors such as different metal components and metal valence states result in variations in the redox properties of the catalyst. This indicator reflects the catalyst's ability to adsorb and activate oxygen, which is fundamental to high catalytic activity. Secondly, controlling the specific morphology of the catalyst can enhance the contact efficiency between the catalyst's active sites and soot particles, which is another crucial factor influencing the catalytic activity. Based on this theory, cobalt‐based oxide materials can be further optimized for catalytic performance by controlling their intrinsic activity and morphology through various means. One strategy involves modifying cobalt‐based catalysts with doping, loading, and solid solution techniques to increase the yield of active oxygen.[^29^, ^30^] Another strategy is to prepare catalysts with specific morphology to enhance the solid‐solid contact efficiency so as to promote the transfer of active oxygen species from catalyst to the soot.[^31^, ^32^] This paper elaborates on the influence of the intrinsic activity and morphology of cobalt‐based oxide catalysts on their catalytic performance and discusses their applications in catalytic soot removal (Figure 2). Finally, the challenges and prospects of cobalt‐based oxide catalysts in catalyzing soot are summarized and discussed.

## 2 INTRINSIC ACTIVITY OF COBALT‐BASED SOOT OXIDATION CATALYSTS

The intrinsic activity of catalyst is an important factor in determining catalytic performance,[^33^, ^34^] as it is related to the adsorption and activation properties of active sites for O_2_ and NO at the metal oxide interface. By selecting the catalyst components, the ability of the redox and active species can be improved, so as to achieve efficient catalytic oxidation of soot. In recent years, researchers have designed and prepared various catalytic oxidation catalysts with excellent intrinsic activity. For example, high‐performance noble metal catalysts such as platinum, silver, and palladium^[35]^; economically viable and low‐toxicity Ce‐based, La‐based, and Pr‐containing rare earth oxide catalysts[^36^, ^37^, ^38^]; and transition metal oxide catalysts with good redox properties and low cost, such as manganese, iron, and copper, have been extensively studied.[^6^, ^39^] Here, we mainly discuss the role of cobalt‐based catalysts in the catalytic oxidation of soot. Researchers have studied methods such as doping, loading, and forming solid solutions to introduce other metals into cobalt‐based catalysts to further enhance the catalytic oxidation activity of the materials.

### 2.1 Pure CoO_*x*_ catalysts

Among various transition metal catalysts, cobalt oxide exhibits relatively high activity and strong redox ability, finding wide applications in fields such as catalytic soot oxidation,[^40^, ^41^] VOCs oxidation,[^42^, ^43^, ^44^] as well as CO and hydrocarbon oxidation.[^45^, ^46^] This is attributed to the fact that cobalt possesses three unfilled d orbitals, which enables it to form neither too strong nor too weak bonds, thereby providing optimal interactions with chemical species reaching the surface, aiding in the adsorption and desorption of reactants and products.^[47]^ Jiang et al.^[41]^ prepared a series of 0D Co_3_O_4_ nanoparticles using different template removal agents, confirming their outstanding efficacy in catalyzing soot oxidation and reducing NO_x_ emissions. Under tight contact conditions, the T_50_ was 286°C. The catalytic oxidation process involved the continuous conversion between Co^3+^ and Co^2+^, with the increased of Co^3+^ notably offering advantageous sites for oxygen adsorption.

Defect engineering is considered as an effective strategy to further optimize the active centers based on the excellent intrinsic activity of cobalt‐based catalysts. Numerous studies have demonstrated that metal defects can provide more active centers for catalysis while the accompanying oxygen vacancies facilitate the replenishment of gaseous oxygen on the catalyst surface. These active oxygen species ($O_{2}^{-}$) on the surface of cobalt‐based catalysts, such as oxygen vacancies and oxygen ions, can adsorb reactants and participate in the activation and conversion processes of substrate oxidation, thereby promoting oxidation reactions and improving the catalytic efficiency and selectivity.[^48^, ^49^] For example, Song et al.^[50]^ used a designed dual‐template method to prepare a defect‐rich Co_3_O_4_. This approach increased the surface area of Co_3_O_4_ and promoted the migration rate of lattice oxygen. As a result, the material exhibited high catalytic activity for low‐temperature CO oxidation. Based on this principle, defect engineering also plays a significant role in catalyzing soot typically achieved through doping with other metals.

In addition to the active oxygen soot oxidation mechanism, Co_3_O_4_ can also accelerate the combustion of soot through the “NO_2_‐assisted combustion mechanism.” During the catalytic oxidation of soot particles, the intermediate product NO can be oxidized to NO_2_ by the catalyst, and NO_2_ exhibits strong oxidizing ability, which can assist in the oxidation of soot particles. This catalytic process is achieved through the repeated conversion and cycling between NO and NO_2_.^[51]^ Previous studies have reported the importance of the efficient cycling of NO↔NO_2_↔soot, with more emphasis on the use of platinum‐based catalysts to assist soot catalysis.^[52]^ However, cobalt‐based catalysts are also effective NO_2_ generators and can efficiently utilize NO_*x*_, even comparable to industrially available platinum‐based catalysts.^[53]^ In the presence of NO_*x*_, the Co_3_O_4_ solid phase acts as a pseudo‐platinum phase and provides new NO_2_ supply along the catalytic bed, thereby accelerating the combustion reaction of soot through the NO_2_‐assisted mechanism. When NO_*x*_ is absent, the combustion of soot is consistent with the intrinsic activity of the material for oxidation‐reduction.^[54]^

In summary, the different oxidation states of cobalt oxide catalysts, active oxygen species, and NO_x_ conversion rate collectively influence their catalytic performance in the oxidation of soot, which is of significant importance for enhancing the catalytic efficiency and selectivity. However, at present, the catalytic performance of cobalt‐based catalysts, especially the low temperature activity, still needs to be further regulated by introducing other metal ions.

### 2.2 Doped cobalt‐based oxide catalysts

The catalytic activity of single transition metal oxides depends largely on the redox properties of the metal itself and can be further enhanced by doping. This modification introduces trace metals as a trace phase into the main phase crystal structure. The difference in ionic radius of the trace phase causes lattice distortion in the main phase leading to lattice expansion and contraction. This distortion results in the formation of more oxygen vacancies promoting the activation of oxygen. Ultimately, this leads to an increase in the catalytic activity of the oxide. Therefore, in catalyst design, the performance of the catalyst is often improved by doping other metal ions. In research, T_10_, T_50_, and T_90_ are commonly used to represent the temperatures at which the PM conversion rates reach 10%, 50%, and 90%, respectively. These values are utilized to evaluate the overall activity of catalysts.

The catalytic oxidation performance of the catalysts varies according to different compositions and component ratios. Common dopants utilized in cobalt‐based catalysts include transition metals, rare earth metals, and alkali metals. For instance, Zou et al.^[55]^ prepared Co‐Ce catalysts with different ratios using the citric acid complexation method, among which the Co_0.93_Ce_0.07_ catalyst exhibited the best catalytic performance with a T_50_ of 370°C. The XRD pattern illustrated that the peak position of Co_1−*x*_Ce_*x*_ samples at Co_3_O_4_ (311) generally underwent negative shifts. This may be attributed to the doping of cerium. Cerium cations were partially incorporated into the lattice of Co_3_O_4_ in Co–Ce mixed oxides, resulting in lattice expansion. This lattice change may lead to increased adsorption and desorption ability of the material to oxygen and enhanced redox ability, thereby facilitating the activation of both surface and bulk lattice oxygen at lower temperatures.

**FIGURE 3:**
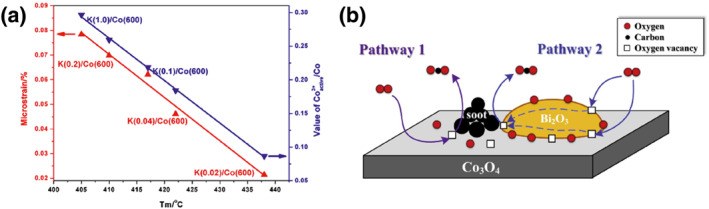
(a) The relationship between (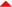) Tm and microstrain and (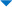) Tm and value of Co^3+^active/Co*.^[56]^ Reproduced with permission.^[56]^ Copyright 2011, Elsevier. (b) Soot oxidation reaction schematic over Bi_x_Co.^[57]^ Reproduced with permission.^[57]^ Copyright 2017, Elsevier.

Moreover, doping involves the addition of metals with lower melting points and higher fluidity, such as alkali metals. This process aims to “wet” the catalyst surface, thereby enhancing the contact between the catalyst and soot. Sun et al.^[56]^ studied the effect of potassium on the catalytic activity of sol‐gel‐prepared Co_3_O_4_ for soot oxidation under loose contact conditions. Potassium, as a promoter, not only facilitated the contact between soot particles and the catalyst but also caused lattice distortion, promoting the formation of active surface Co^3+^ and active O^−^, enhancing the activity of lattice oxygen. From the observation in Figure 3a, it is evident that the augmentation of active Co^3+^ content correlates with the rise of K concentration, indicating that the deposition of K fosters the generation of more active Co^3+^. The findings underscore that the deposition of 4.4 wt.% K significantly enhances the catalytic prowess of Co_3_O_4_ in facilitating soot oxidation under conditions of loose contact, leading to a notable reduction in the maximum oxidation rate temperature (T_m_) of soot from 490°C to 417°C.

So far, a large number of studies have reported the doping of transition metals, rare earth metals, alkali metals, and other substances into Co_3_O_4_ to prepare cobalt‐based soot catalysts. In addition, doping with other metals is also commonly reported. For example, Shang et al.^[57]^ found that bismuth‐modified Co_3_O_4_ (Bi/Co = 0.2) exhibited the highest activity, with T_50_ values of 332°C and 475°C under tight and loose contacts, respectively. Bismuth doping facilitated the formation of oxygen vacancies, accelerated the migration rate of lattice oxygen species, and improved the efficiency of oxygen transport from the gas phase to the soot/catalyst contact point (Figure 3b).

### 2.3 Loaded cobalt‐based oxide catalysts

**FIGURE 4:**
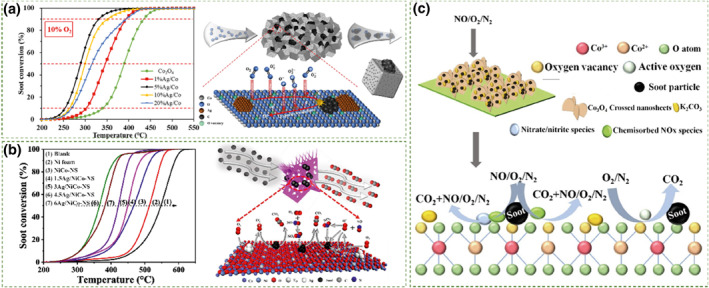
(a) Soot conversion versus temperature in 10% O_2_/N_2_ and Illustration of reaction mechanisms for soot oxidation over the Ag/Co_3_O_4_ catalysts.^[63]^ Reproduced with permission.^[63]^ Copyright 2021, ACS Publications. (b) Soot catalytic combustion activity of different catalysts in O_2_ balanced by N_2_ and Illustration of reaction mechanisms for soot oxidation over the xAg/NiCo‐NS catalysts.^[64]^ Reproduced with permission.^[64]^ Copyright 2022, Elsevier. (c) Illustration of soot oxidation mechanisms over the KCo‐NS catalyst.^[65]^ Reproduced with permission.^[65]^ Copyright 2021, Elsevier.

Surface loading is primarily used for noble metals and alkali metals, which respectively enhance the catalyst's low‐temperature activity and fluidity. Noble metals have received widespread attention in the catalytic field due to their excellent catalytic activity and selectivity. To control production costs, trace loading of noble metals on the catalyst surface is often chosen. This method not only effectively controls production costs but also significantly enhances the dispersion of metal active sites. Additionally, loading noble metals on the catalyst surface also creates numerous dangling and unsaturated bonds, increasing surface defects and effectively improving the catalytic activity. Commonly used noble metals for loading include Ag, Pt, Pd, and Au.[^58^, ^59^, ^60^, ^61^] Among them, Ag has been extensively studied for its excellent performance and relatively low cost as well as its interaction with the support.^[62]^ Chen et al.^[63]^ prepared a series of Ag/Co_3_O_4_ catalysts with different silver loading levels using the impregnation method. Among them, the 5% Ag/Co_3_O_4_ catalyst exhibited competitive catalytic activity for soot oxidation, with a T_50_ below 290°C under 10% O_2_/N_2_ and tight contact conditions, as shown in Figure 4a. The increase in catalytic activity caused by Ag loading is mainly attributed to two reasons. First, the partial incorporation of Ag^+^ species into the Co_3_O_4_ lattice leads to lattice expansion and the formation of defect structures, further enhancing the interaction at the Ag‐Co_3_O_4_ interface. Moreover, the specific surface area of the Ag‐loaded Ag‐Co_3_O_4_ catalyst increased, which provided more active sites for soot oxidation. Yang et al.^[64]^ designed and synthesized highly active catalysts loaded with Ag (XAg/NiCo‐NS). With a T_50_ of 333°C for the 4.5Ag/NiCo‐NS sample, which was 50°C lower than that of the pure NiCo‐NS catalyst without Ag loading. As shown in Figure 4b, this is because the metal‐support interaction between Ag and NiCo‐NS weakened the Co‐O bonds, increased the number of surface oxygen vacancies, and improved the intrinsic activity determined by the catalyst's turnover frequency (TOF). Zou et al.^[66]^ successfully prepared xAg/Co_0.93_Ce_0.07_ composite oxides via citric acid complexation method. Under tight contact condition, T_10_ was 197°C. According to the experimental results, Ag in the composite metal system plays several roles. Firstly, Ag loading leads to the formation of silver oxide on the sample surface and promotes active oxygen generation. Additionally, the low melting point of Ag also promotes its mobility, thus enhancing the contact between soot and the catalyst. Furthermore, silver oxides aid in the adsorption of reactants, forming crucial complex π bonds needed for peroxide and superoxide species formation, thereby promoting soot oxidation at low temperatures.^[67]^

The introduction of other transition metal oxides to construct non‐noble metal‐loaded cobalt‐based catalysts can also play a role in improving catalytic activity. For example, Alkali metals such as potassium have low melting points and sufficient mobility to improve the contact efficiency between soot and catalyst.[^68^, ^69^] Cao et al.^[65]^ found that the KCo‐NS catalyst loaded with potassium showed good catalytic activity in soot oxidation, achieving a T_50_ of 333°C under loose contact mode (Figure 4c). This is because potassium loading increased the number of oxygen vacancies on the catalyst surface while enhancing the low‐temperature redox capability of the sample. Moreover, at high temperatures, potassium species can improve the surface migration rate to enhance the contact efficiency between potassium, including the catalyst, and soot particles.

### 2.4 Solid solution cobalt‐based oxide catalysts

It has been reported that many researchers often modify catalysts through solid solutions to introduce other metal ions.^[70]^ Unlike doping, the amount of the solid solution phase can be substantial or even unlimited. These metal ions enter the lattice of the modified metal but usually do not alter the lattice structure. Such changes are often undetectable by conventional methods. Currently, research on cobalt‐based solid solutions is limited and mainly used in areas such as hydrogen production,^[71]^ VOCs catalytic oxidation,^[72]^ and oxygen evolution reaction.^[73]^ Alternatively, some studies directly utilize Co to modify the main phase catalyst to increase the utilization rate of NO in soot oxidation. For example, Li et al.^[74]^ prepared a Ce_0.85_Co_0.15−3*x*_O_2−δ_ solid solution and found that the Ce‐Co catalyst contained surface free Co_3_O_4_ species. These surface‐free Co_3_O_4_ species and Ce_0.85_Co_0.15−3*x*_O_2−δ_ solid solution collectively enhance the catalytic performance of the catalyst, with the former providing NO adsorption centers and the latter promoting oxygen activation, thereby improving the catalytic activity of the catalyst. In addition, Liu et al.^[75]^ synthesized a Co‐SnO solid solution, further demonstrating that the addition of Co significantly increased the content of active oxygen in the catalyst and improved its catalytic performance.

### 2.5 Cobalt‐based oxide catalysts of complex mineral type

**FIGURE 5:**
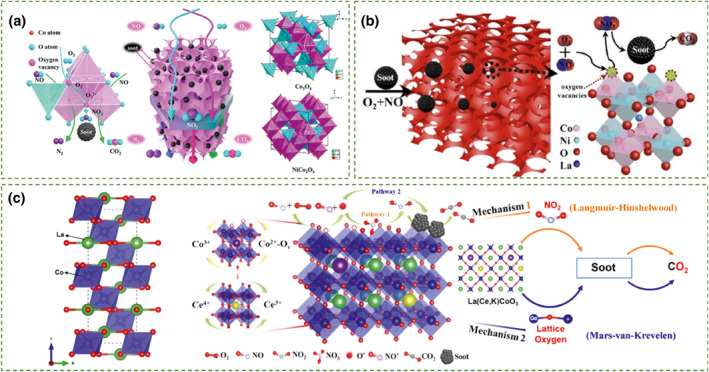
(a) Schematic illustration of 3DOM cobalt‐based spinel catalysts.^[77]^ Reproduced with permission.^[77]^ Copyright 2019, ACS Publications. (b) Reaction mechanism diagram of 3DOM double perovskite‐type La_2_NiCoO_6_ catalysts for soot oxidation.^[78]^ Reproduced with permission.^[78]^ Copyright 2020, Elsevier. (c) LaCoO_3_ unit cell and the schematic illustration of bifunctional effects of the as‐prepared catalysts in soot catalytic oxidation.^[79]^ Reproduced with permission.^[79]^ Copyright 2022, ACS Publications.

Spinel (AB_2_O_4_) and perovskite (ABO_3_) composite oxides are also commonly employed in the catalytic oxidation of diesel engine soot particles. The spinel structure (AB_2_X_4_) contains tetrahedral coordination ${\left(A2+‐X\right)}_{T_{d}}$ and octahedral coordination ${\;\left(B3+‐X\right)}_{O_{h}}$, making it a typical multi‐coordinated catalyst configuration.^[76]^ The catalytic activity of this material mainly depends on the properties of the metal ions at the A and B sites as well as the doping ions substituting A and B. Different metal ion doping can alter the lattice structure of Co_3_O_4_ (cation substitution effect), thereby increasing the density of oxygen vacancies and lattice oxygen migration rate. Zhao et al.^[77]^ demonstrated the enhancement of soot oxidation performance of cobalt‐based catalysts by transition metal doping. They prepared cobalt‐based spinel catalysts M_x_Co_3−*x*_O_4_ using carboxy‐modified colloidal crystal templating method (CMCCT), where Co was in the B position and M (Zn or Ni metal) was in the A position. This study mainly investigated the effect of A‐site changes on catalyst performance. Through a directed substitution strategy of Ni metal ions, they effectively increased the density of oxygen vacancies in Co_3_O_4_. This resulted in an improved migration rate of lattice oxygen leading to high catalytic activity for soot oxidation under loose contact condition. The T_50_ of NiCo_2_O_4_ was 379°C, which was significantly reduced by 213°C compared to pure soot. Additionally, when NO approached the catalyst surface, the strong oxidation capability of Co_3_O_4_ and abundant surface‐active oxygen could effectively oxidize it to NO_2_, significantly enhancing the catalytic performance of soot oxidation (Figure 5a).

Recent studies have also shown that perovskite‐type oxides with A or B ions in different oxidation states and special oxygen storage properties are conducive to improve the catalytic activity of oxidation reactions. Substitution of A or B ions in ABO_3_ with other metal ions is also an important research focus in the field of soot combustion. Perovskite is mainly composed of rare earth element A‐site ions coordinated with 12 oxygen ions, and transition metal element B‐site ions coordinated with 6 oxygen ions, located at the center of [BO_6_] octahedra. In deep oxidation reactions, B‐site ions serve as active centers which are crucial for enhancing catalytic performance. Mei et al.^[78]^ prepared La_2_NiB'O_6_ (B' = Mn, Fe, Co, Cu) catalysts with a three‐dimensional ordered macroporous structure, among which La_2_NiCoO_6_ catalyst exhibited the highest catalytic activity for soot oxidation, with a T_50_ of 362°C under loose contact conditions, approaching the catalytic performance of platinum‐based catalysts. As shown in Figure 5b, this was mainly attributed to the synergistic effect of Ni and Co ions promoting the oxidation of NO to NO_2_, which was a key step in catalyzing soot. Wang et al.^[80]^ conducted a comparative study of nanoscale cobalt, manganese, and iron‐based perovskite‐type composite oxide catalysts, confirming the good catalytic performance of cobalt‐based perovskite‐type oxides in simultaneously eliminating soot particles and NO. They can significantly reduce the combustion temperature of soot particles and increase the conversion rate of NO. Furthermore, Yu et al.^[79]^ studied the substitution of A‐site Co metal with alkali metal K and Ce ions, obtaining La_0.9_Ce_0.05_K_0.05_CoO_3_ catalysts with a large number of CO_2_^+^‐O_v_ active centers on the surface and improved oxygen storage capacity through the redox cycle between Ce^4+^ and Ce^3+^. Compared with other catalysts, La_0.9_Ce_0.05_K_0.05_CoO_3_ catalyst exhibits stronger NO adsorption, storage, and oxidation to NO_2_ capability. As shown in Figure 5c, the introduction of Ce and K forms a modified La‐cobalt‐based perovskite catalyst, playing a dual role in promoting soot catalytic oxidation. The increased presence of more active oxygen species formed by oxygen vacancies and surface lattice oxygen consumption and replenishment, as well as the dynamic interaction of NO_x_ species formed on the catalyst surface, thereby enhances the catalytic activity of K and Ce‐modified La‐cobalt‐based perovskite catalysts.

## 3 COBALT‐BASED OXIDE CATALYSTS OF SPECIFIC MORPHOLOGY AND STRUCTURE

**FIGURE 6:**
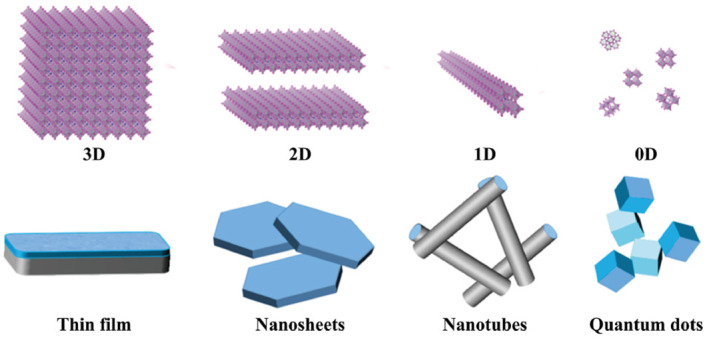
Structure diagram of the 3D to 0D catalyst.^[83]^ Reproduced with permission.^[83]^ Copyright 2020, Wiley‐VCH GmbH.

Controlling the morphology of catalysts is just as crucial as their intrinsic catalytic activity for enhancing overall performance. Normally, gaps between catalyst particles hinder direct contact with soot, limiting active site utilization. By optimizing the morphology, the contact efficiency between soot and catalysts increases, maximizing the surface area and active oxygen generation for better soot oxidation.[^81^, ^82^] Therefore, effective soot oxidation catalysts must have a high specific surface area and unique structures to optimize reactant‐catalyst interfaces. Currently, much research is focused on designing catalysts with different morphologies to achieve better contact efficiency for soot oxidation. The catalytic performance of catalysts strongly depends on their morphology. As illustrated in Figure 6, nanomaterials can be categorized into zero‐dimensional (0D) structures, one‐dimensional (1D) structures, two‐dimensional (2D) structures, as well as three‐dimensional (3D) structures. Materials with similar compositions but different dimensions often exhibit unique features, such as a high density of active sites for 0D materials, porous structures for 1D materials, tunable defects for 2D materials, and multiscale porosity for 3D materials.^[83]^

### 3.1 Zero‐dimensional structure

0D structures, such as quantum dots, clusters, and nanospheres, constitute 0D catalysts, which are assemblies of nanometer‐sized particles. These catalysts typically exist in powder form and offer advantages such as high surface area, abundant active sites, and high catalytic efficiency. Due to their intrinsic structural characteristics, 0D catalysts possess more adsorption and active edge sites per unit mass.^[84]^ For instance, Wan et al.^[85]^ designed a single‐atom cobalt catalyst, which exhibited significantly enhanced electrocatalytic activity and selectivity for the oxygen reduction reaction, approaching that of benchmark Pt‐based catalysts. However, in high‐temperature catalytic reactions, 0D catalysts such as metal nanoclusters tend to undergo severe sintering due to the sharp increase in surface energy with decreasing particle size. This unavoidable sintering leads to the loss of active metal surface area, thereby causing catalyst deactivation. Based on a series of studies, it has been found that catalysts with enlarged particle spacing demonstrate superior catalytic stability.^[86]^ Therefore, in catalytic oxidation research, 0D catalysts are usually combined with other structured materials so that it could exert the advantages of the 0D structure while enlarging the particle spacing. For example, to enhance the efficacy of cobalt‐based oxide catalysts, the active phases are commonly dispersed onto carriers characterized by a high specific surface area. Grazybek et al.^[87]^ utilized porous structures and high surface area zeolites as carriers for cobalt metal nanoparticles, fully exploiting the effects of cobalt metal and alkali doping in catalyzing soot oxidation. Although the zeolite carriers used in the application are completely devoid of catalytic activity, catalysts represented by zeolite carriers have been successfully developed.^[88]^ This approach can address the deactivation problem caused by the sintering of cobalt nano‐clusters and leverage the catalytic benefits of 0D structure catalysts.

### 3.2 One‐dimensional structure

**FIGURE 7:**
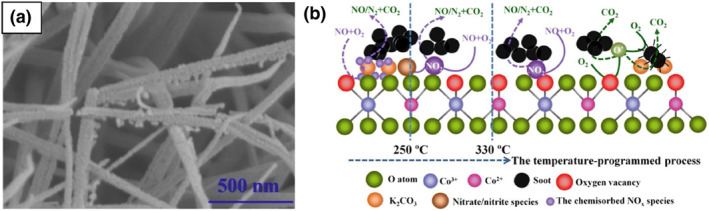
(a) SEM images of the different catalysts: the fresh 5KCo‐NW. (b) Illustration of reaction mechanisms for soot oxidation over the xKCo‐NW catalysts under gravitation contact mode (GCM).^[91]^ Reproduced with permission.^[91]^ Copyright 2017, Elsevier.

One‐dimensional structures mainly include nanowires, nanofibers, and nanorods. Compared with 0d structural materials, 1d structural materials retain the small size effect (diameter nanometer) of 0d structure while reducing the possibility of particle aggregation‐induced deactivation. This enhancement can be achieved simply by controlling the lattice exposed plane, thus significantly improving the adsorption and activation of reactants and enhancing the catalytic efficiency and reaction rates.^[89]^ Ma et al.^[90]^ synthesized nanorods of cobalt oxide that mainly exposed the catalytically active (110) lattice plane. This plane typically contains the Co^3+^ ions responsible for catalytic oxidation reactions. Consequently, these nanorods exhibited many active sites, which improved the catalytic activity of Co_3_O_4_. Furthermore, the open spaces among the one‐dimensional structures helped the deposition of soot particles. This also contributed to the improved catalytic efficiency. Cao et al.^[91]^ synthesized potassium‐promoted Co_3_O_4_ nanowires on three‐dimensional macroporous Ni foam (xKCo‐NW) using a simple hydrothermal and impregnation method. Figure 7a illustrated the SEM of Co_3_O_4_ nanowires, the presence of open macropores between these nanowires would enhance the deposition of soot particulates. These catalysts exhibited excellent catalytic activities for soot oxidation under gravitation contact mode, particularly the 5KCo‐NW catalyst. As shown in Figure 7b, under an O_2_/N_2_ atmosphere, soot catalytic oxidation on this catalyst followed the oxygen spillover mechanism, leading to the generation of gaseous NO_2_ and achieving T_50_ at 324°C. Yang et al.^[92]^ synthesized cactus‐like Co_3_O_4_/OMS‐2 nanorods with abundant pores, providing sufficient macroporous space to enhance the contact efficiency between soot and the catalyst. Compared with traditional powder‐coated catalysts, the consumption of the synthesized catalyst was reduced by ∼13 times with the same thickness, and the contact area was increased by ∼4 times with the same weight.

### 3.3 Two‐dimensional structure

**FIGURE 8:**
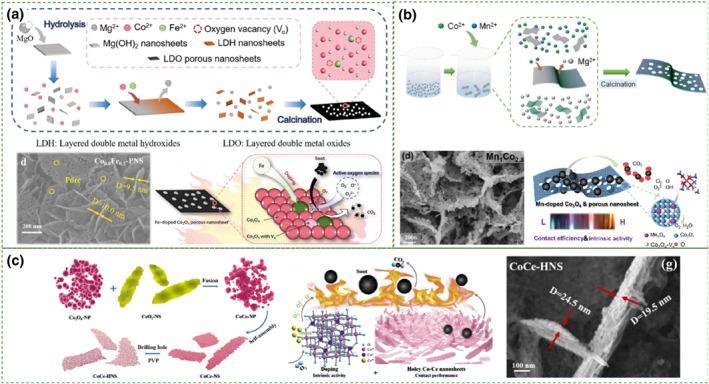
(a) Schematic illustration of the synthesis process of Co_x_Fe_1−*x*_‐PNS, SEM images of Co_0.8_Fe_0.2_‐PNS, and schematic diagram of catalytic soot reaction mechanism.^[94]^ Reproduced with permission.^[94]^ Copyright 2022, Elsevier. (b) Preparation of catalyst by magnesium oxide template replacement method, diagrams of the reaction mechanism of the intrinsic activity and contact efficiency for catalytic soot oxidation, SEM images of Mn_1_Co_2.3_.^[95]^ Reproduced under terms of the CC‐BY license.^[95]^ Copyright 2024, Miaomiao Hu, published by [KeAi]. (c) Schematic diagram of Co‐Ce catalyst synthesis, schematic diagram of catalytic soot reaction mechanism, SEM images of CoCe‐HNS.^[96]^ Reproduced with permission.^[96]^ Copyright 2020, Elsevier.

Two‐dimensional materials (including nanosheets and ribbons) are relatively thin in the third dimension, exposing more surface atoms compared to bulk materials. They exhibit a higher utilization rate of active sites in catalytic reactions and possess larger external surface areas. Therefore, based on intrinsic activity control, two‐dimensional nanosheets have certain advantages in enhancing solid‐solid contact efficiency. Leveraging the advantages of two‐dimensional nanomaterials, transforming conventional bulk catalysts into two‐dimensional nanosheets and applying them in soot particle combustion may enhance the catalytic performance. Shi et al.^[93]^ conducted in‐situ etching of the La layer of LaMnO_3_ to obtain ultrathin MNO_2−*x*_ nanosheet arrays. Under loose contact conditions, the ignition temperature (T_10_) was 342°C, while with strong oxidizing NO_x_‐assisted catalysis under loose contact conditions, T_10_ was successfully lowered to 200°C. The morphology of nanosheets provided a larger external surface area, resulting in higher contact efficiency between soot particles and the catalyst. However, due to the non‐layered nature of cobalt‐based catalysts, it is challenging to obtain ultrathin nanosheets through conventional catalyst preparation methods or physical or chemical stripping methods. Therefore, some studies have produced Co_3_O_4_ nanosheets by making nanosheet sacrificial templates. Li et al.^[94]^ synthesized a series of Co‐Fe oxides with nanosheet‐like structures for soot oxidation using a magnesium oxide‐mediated method (Figure 8a). The 2D nanosheet structure of Co‐Fe oxides provided a larger external surface area, and its porous structure facilitated gas adsorption and diffusion. Among them, Co_0.8_Fe_0.2_‐PNS exhibited the best performance, characterized by the lowest activation energy (114 kJ mol^−1^) and the highest oxygen desorption capacity (37.76 mmol g^−1^). Under loose and tight contact conditions, its T_50_ values were 363°C and 305°C respectively. Using the same method, Hu et al.^[95]^ prepared a series of Mn_*x*_Co_*y*_ nanosheet catalysts with internal mesopores (Figure 8b). In terms of catalytic performance, Mn_1_CO_2.3_ exhibited the highest catalytic activity, with a T_50_ of 363°C under loose contact conditions. This was attributed to the higher contact efficiency of the nanosheets and their strong adsorption capability for gaseous reactants.

According to the idea of increasing the specific surface area to enhance the catalytic efficiency, if two‐dimensional nanosheets possess abundant porous structures, they may further improve the performance of soot particle catalytic combustion. For example, Cui et al.^[96]^ prepared CoCe‐HNS mesoporous nanosheets for soot particle catalytic combustion using a polyvinylpyrrolidone (PVP)‐assisted hydrothermal method (Figure 8c). The two‐dimensional mesoporous sheet structure showed better solid‐solid contact efficiency at the three‐phase boundary of soot oxidation reactions compared to CoCe‐NS nanosheets. Under loose contact conditions without NO_x_ ‐assisted catalysis, the T_10_ and T_50_ of this catalyst were 322 and 367°C, respectively. Xing et al.^[97]^ prepared Co_3_O_4_/CeO_2_ nanosheets (Co/Ce‐NS) using a sequential hydrothermal and impregnation method. The hierarchical mesoporous and macroporous nanosheets increased the contact area during soot oxidation and facilitated the adsorption of gaseous reactants. Among various morphologies, nanosheets containing mesoporous materials exhibited higher solid‐solid contact efficiency and gas adsorption and diffusion due to their larger external surface area and mesopores, making them a promising choice for soot oxidation.

### 3.4 Three‐dimensional structure

**FIGURE 9:**
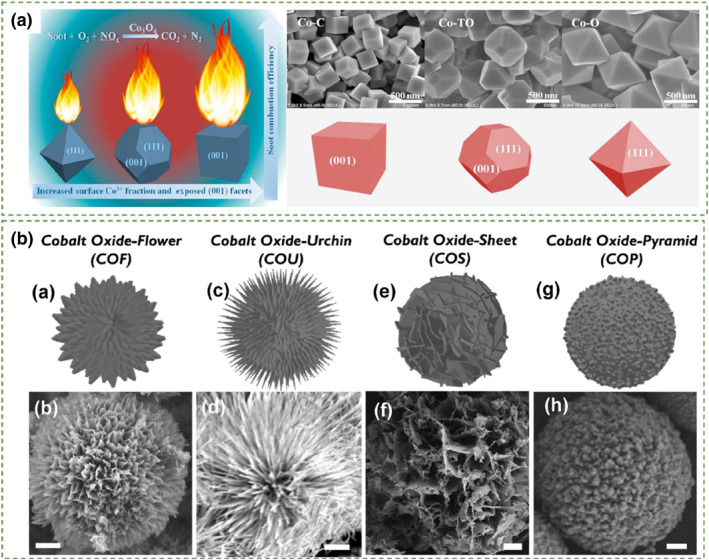
(a) FESEM images and activity comparison of different catalysts.^[99]^ Reproduced with permission.^[99]^ Copyright 2018, Elsevier. (b) Illustrations and SEM pictures of various HCOMs (the scale bar is 1 μm).^[100]^ Reproduced with permission.^[100]^ Copyright 2020, Elsevier.

Common three‐dimensional materials include bulk nanomaterials and three‐dimensionally ordered macroporous (3DOM) materials categorized based on their special morphology. The main characteristic of three‐dimensional porous materials is the highly developed pore structure, which may include macropores (diameter >50 nm), mesopores (2 < diameter <50 nm), and/or micropores (diameter <2 nm).^[98]^ Due to advantages such as tunable pore sizes and large specific surface areas, three‐dimensional structural catalysts have been widely investigated as promising catalysts for soot oxidation. Adjusting the crystal facets of Co_3_O_4_ to form specific morphologies can enhance the catalytic activity of Co_3_O_4_. For example, Zhai et al.^[99]^ synthesized 3d nanocubes Co_3_O_4_ with (001) crystal facets. Due to their higher content of active Co^3+^ sites, surface lattice oxygen, and better redox capability, they displayed significantly higher catalytic efficiency in soot oxidation compared to Co_3_O_4_ octahedrons enclosed by (111) facets and Co_3_O_4_ truncated octahedrons with co‐exposed (001) and (111) facets, respectively. This enhancement was mainly attributed to the chemical nature of the exposed (001) facets (Figure 9a).

Tsai et al.^[100]^ prepared four different morphologies of Co_3_O_4_ catalysts, including Co_3_O_4_ with flower morphology (COF), Co_3_O_4_ with urchin morphology (COU), Co_3_O_4_ with sheet morphology (COS), and Co_3_O_4_ with pyramid morphology (COP), to compare their respective catalytic activities (Figure 9b). Overall, these materials exhibited high specific surface areas and porous nanostructures leading to significantly higher catalytic activity compared to commercial spherical Co_3_O_4_ nanoparticles and most of the previously reported cobalt‐based catalysts. Particularly, COP demonstrated superior catalytic activity compared to other morphologies, primarily due to its higher content of active surfaces and better redox properties, making it most favorable for soot oxidation. Specifically, among these morphologies, COP showed the most favorable characteristics for soot oxidation, attributed to its higher content of active surfaces and superior redox properties, which accelerate the oxidation of soot.

**FIGURE 10:**
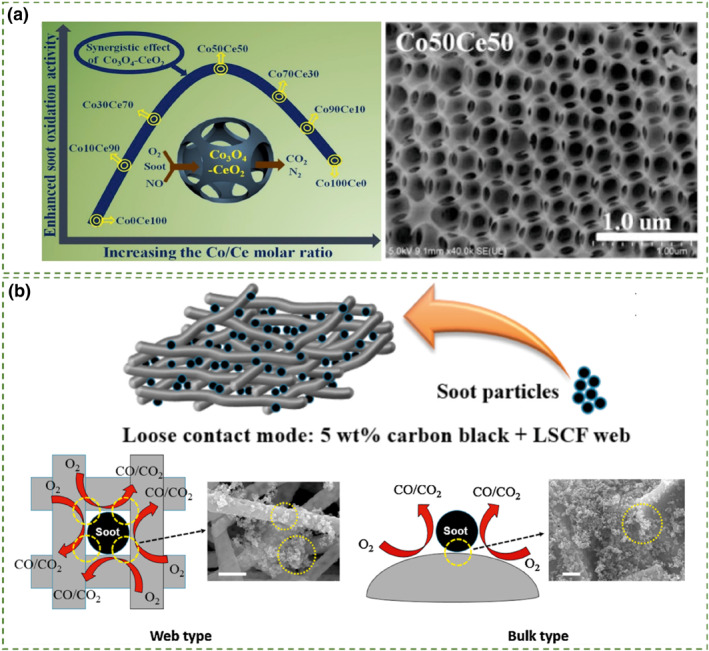
(a) The activity comparison of catalysts with different Co‐Ce ratios and their FESEM images.^[107]^ Reproduced with permission.^[107]^ Copyright 2019, Elsevier. (b) Schematic illustration of the soot oxidation process and surface SEM images.^[108]^ Reproduced with permission.^[108]^ Copyright 2016, Elsevier.

Most studies have focused on preparing high surface area porous catalysts. However, for traditional porous catalysts, only the external surface area can be effectively utilized for catalyzing soot oxidation, as the pore sizes are smaller than the average size of soot particles (>25 nm), hindering the entry of soot particles into the pores and contact with the internal surface.[^5^, ^101^, ^102^] Three‐dimensional ordered macroporous (3DOM) catalysts feature interconnected large pores (>50 nm), providing pathways for soot particles to traverse through the catalyst interior.[^103^, ^104^] According to previous reports, catalysts with open large pores facilitate contact between the catalyst and soot particles, greatly enhancing the catalytic performance in soot oxidation.^[105]^ The periodic arrangement of large pores in 3DOM catalysts significantly reduces the transfer resistance of soot particles within the pores, further enhancing the catalytic activity. The formation of the 3DOM structure involves sacrificial templating and precursor interactions. Duan et al.^[106]^ used self‐assembled PS latex spheres to prepare templates which were impregnated and calcined with various metal precursors (Ce, Ni, Mg, Cu, Zn, Co). The Ce_0.5_Ni_0.1_Mg_0.1_Cu_0.1_Zn_0.1_Co_0.1_O_x_ catalyst with ordered macropores, active oxygen species, and high entropy‐stabilized structure exhibited superior activity (T_50_ = 393°C) in soot oxidation under harsh conditions (4.2 vol% moisture, 20 ppm SO_2_), surpassing that of the sol–gel control sample (T_50_ = 419°C), 3DOM‐CeO_2_ (T_50_ = 506°C), commercial CeO_2_ (T_50_ = 519°C), and 1% Pt/Al_2_O_3_ (T_50_ = 595°C). Likewise, Zhai et al.^[107]^ prepared Co/Ce molar ratio‐controllable three‐dimensional ordered macroporous Co_3_O_4_‐CeO_2_ catalysts using a colloidal crystal templating method, which were applied in NO_x_ ‐assisted soot oxidation reactions, exhibiting high soot oxidation activity (Figure 10a). This is attributed to the synergistic promotion effect of the 3DOM structure on the Co_3_O_4_‐CeO_2_ catalyst, which enhances the contact interface between soot and the catalyst. Simultaneously, both Co_3_O_4_ and CeO_2_ exhibit high NO_*x*_ storage capacity and oxidation ability, thus synergistically facilitating the process. To increase the contact area between the catalyst and soot, research has also explored shape‐controlled network structures, such as Lee et al.^[108]^ fabricated the La_1−*x*_Sr_*x*_CO_2_Fe_8_O_3−δ_ perovskite fibrous webs with different Sr doping levels. Using an electrospinning technique to achieve a unique matrix morphology with a hierarchical porous structure. As shown in Figure 10b, compared to the bulk type, the web‐type mesh structure of the web type offers increased contact area and 3D internal space, enabling more effective capture of soot particles. Well‐distributed pore sizes and perfectly induced three‐dimensional ordered porous nanostructures can enhance the contact efficiency of soot‐catalyst interactions by promoting mass transfer and diffusion of soot particles, and 3DOM catalysts exhibit higher activity in soot oxidation compared to disordered macroporous and powder catalyst.[^27^, ^109^]

## 4 SUMMARY AND PROSPECTS

Developing catalysts with low‐temperature activity, low cost, and high stability is the core issue for the removal of diesel engine exhaust PM. Considering the excellent catalytic performance and cost‐effectiveness of cobalt‐based catalysts, this review discusses the latest research achievements of cobalt‐based catalysts in soot catalysis, focusing on the design and improvement of the intrinsic activity and morphology structure of cobalt‐based catalysts to enhance their catalytic oxidation performance towards soot.

Specifics are shown in Table 1, from the perspective of modification, preparing composite mineral materials or loaded cobalt‐based catalysts can promote catalytic reactions, with their T_10_ mostly below 300 under loose contact mode. Analyzing from the aspect of morphology control, catalysts with 1D and 2D structures exhibit lower catalytic temperatures. Particularly, 2D mesoporous structures possess larger specific surface areas, significantly enhancing catalysis. In practical catalyst design, integrating the advantages of both strategies is feasible. For example, preparing a cobalt‐based catalyst with a 2D structure loading may hold certain research prospects. Despite the considerable efforts and progress made, challenges still exist in the preparation and application of cobalt‐based oxide catalysts.

(i) Numerous synthesis techniques have emerged for producing cobalt‐based oxide catalysts with exceptional catalytic efficacy. Nevertheless, the majority of these methods are complex, necessitating costly raw materials and precise templates to regulate metal ratios, structure, and morphology. Consequently, the pursuit of a universally applicable method to address these challenges while upholding high efficiency remains an ongoing obstacle for cobalt‐based oxide catalysts.
(ii) The composition of diesel PM is indeed intricate, comprising various elements such as soot particles, soluble organic matter (SOF), sulfate, ash, and other diverse constituents. However, the capability of most catalysts is confined to targeting specific pollutants among complex compound composition, posing a challenge to achieve comprehensive control over diesel PM.
(iii) While fundamental research has showcased the remarkable catalytic prowess of cobalt‐based oxide catalysts in mitigating pollutants like soot, their practical application under rigorous emission standards presents challenges. Many catalysts encounter difficulty in entirely eradicating pollutants in real‐world scenarios. Sustaining the exemplary catalytic performance of cobalt‐based oxide catalysts thus poses a persistent challenge, underscoring the need for additional research to bridge the gap between theoretical findings and practical implementation.

**TABLE 1:** Review on the catalytic performance of cobalt‐based catalysts for the elimination of soot particles.

Design	Sample	Catalyst: Soot	Reaction conditions	T_10_/T_50_/T_90_ (°C)		References
				Loose	Tight	
Pure CoO_x_	m‐Co_3_O_4_‐Na	1:19	25 mL min^−1^, 5% O_2_+ 2000 ppm NO+ He		250/286/‐	[41]
Doped	Co_0.93_Ce_0.07_	1:19	50 mL min^−1^, air		315/370/407	[55]
	K (0.1)/Co (600)	1:9	100 mL min^−1^, 8% O_2_+ Ar	‐/417/‐		[56]
	Bi_0.2_Co	1:9	100 mL min^−1^, 8% O_2_+ Ar	379/475/546	281/332/360	[57]
Loaded	5% Ag/Co	1:10	100 mL min^−1^, 21% O_2_+ N_2_		247/280/323	[63]
	xAg/Co_0.93_Ce_0.07_	1:19	25 mL min^−1^, 5% O_2_+ 2000 ppm NO+ He		197/−/−	[66]
	4.5 Ag/NiCo‐NS	1:20	100 mL min^−1^, 10% O_2_+ 500 ppm NO+ N_2_	269/333/‐		[64]
	KCo‐NS	1:10	100 mL min^−1^, 10% O_2_+ 600 ppm NO+ N_2_	229/333/‐		[65]
Solid solution	8% Cs/SC85‐15	1:10	30 mL min^−1^, 10% O_2_+ Ar	335/‐/460		[75]
Complex mineral type	NiCo_2_O_4_	1:10	300 mL min^−1^, 5% O_2_+ 1000 ppm NO+ Ar	308/379/423		[77]
	La_2_NiCoO_6_	1:10	50 mL min^−1^, 5% O_2_+ 0.2% NO+ 5% H_2_O+ Ar	288/362/412		[78]
	La_0.90_K_0.10_CoO_3_	1:5	50 mL min^−1^, 5% O_2_+ 2000 ppm NO+ He	298/398/463		[80]
	La_0.9_Ce_0.05_K_0.05_CoO_3_	1:10	50 mL min^−1^, 5% O_2_+ 0.2% NO+ Ar	273/306/327		[79]
0 D	6K Co/FER	1:10	60 mL min^−1^, 10% O_2_+ 0.33% NO+ He	−/−/450		[87]
1 D	5KCo‐NW	1:10	100 mL min^−1^, 5% O_2_+ 600 ppm NO+ N_2_	279/324/−		[91]
	8Co/Mn‐NR	1:10	100 mL min^−1^, 10% O_2_+ 600 ppm NO+ 10% H_2_O+ N_2_	330/378/−		[92]
2 D	Co_0.8_Fe_0.2_‐PNS	1:10	50 mL min^−1^, 5% O_2_+ Ar	302/363/403	240/305/351	[94]
	Mn_1_Co_2.3_	1:10	30 mL min^−1^, 10% O_2_+ 500 ppm NO+ Ar	290/363/408		[95]
	CoCe‐HNS	1:10	30 mL min^−1^, 5% O_2_+ Ar	‐/367/422	‐/311/352	[96]
	0.6Co/Ce‐NS	1:5	100 mL min^−1^, 10% O_2_+ 600 ppm NO+ N_2_ or 10% O_2_	319/380/‐		[97]
3 D	Co‐C	1:10	80 mL min^−1^, 5% O_2_+ 0.25% NO+ N_2_	‐/421/‐		[99]
	COP	1:9	100 mL min^−1^, 20.5% O_2_+ N_2_	327/361/396		[100]
	Co_50_Ce_50_	1:10	80 mL min^−1^, 5% O_2_+ 0.25% NO+ N_2_	‐/406/‐		[107]
	LSCF‐6428‐web	1:20	100 mL min^−1^, 6% O_2_+ 3% H_2_O+ N_2_	397/‐/647		[108]

## AUTHOR CONTRIBUTIONS

The manuscript was written through the contributions of all authors. All authors have given approval to the final version of the manuscript.

## CONFLICT OF INTEREST STATEMENT

The authors declare no conflicts of interest.

## Data Availability

Data sharing is not applicable to this article as no new data were created or analyzed in this study.
